# Facile production of nanocomposites of carbon nanotubes and polycaprolactone with high aspect ratios with potential applications in drug delivery†

**DOI:** 10.1039/c7ra13553j

**Published:** 2018-05-04

**Authors:** Edyta Niezabitowska, Jessica Smith, Mark R. Prestly, Riaz Akhtar, Felix W. von Aulock, Yan Lavallée, Hanene Ali-Boucetta, Tom O. McDonald

**Affiliations:** Department of Chemistry, University of Liverpool Crown Street Liverpool L69 7ZD UK Thomas.Mcdonald@liverpool.ac.uk; Department of Mechanical, Materials and Aerospace Engineering, School of Engineering, University of Liverpool Brownlow Hill Liverpool L69 3GH UK; School of Environmental Sciences, University of Liverpool Jane Herdman Building, Brownlow Street Liverpool L69 3GP UK; The School of Pharmacy, College of Medical and Dental Sciences, University of Birmingham Edgbaston Birmingham B15 2TT UK

## Abstract

The geometries and surface properties of nanocarriers greatly influence the interaction between nanomaterials and living cells. In this work we combine multiwalled carbon nanotubes (CNTs) with poly-ε-caprolactone (PCL) to produce non-spherical nanocomposites with high aspect ratios by using a facile emulsion solvent evaporation method. Particles were characterised by dynamic light scattering (DLS), scanning electron microscopy (SEM), atomic force microscopy (AFM) and asymmetric flow field flow fractionation (AF4). Different sizes and morphologies of nanoparticles were produced depending on the concentration of the sodium dodecyl sulphate (SDS), CNTs and PCL. Rod-like PCL-CNT nanostructures with low polydispersity were obtained with 1.5 mg mL^−1^ of SDS, 0.9 mg mL^−1^ of CNTs and 10 mg mL^−1^ PCL. AFM analysis revealed that the PCL and PCL-CNT nanocomposite had comparatively similar moduli of 770 and 560 MPa respectively, indicating that all the CNTs have been coated with at least 2 nm of PCL. Thermogravimetric analysis of the PCL-CNT nanocomposite indicated that they contained 9.6% CNTs by mass. The asymmetric flow field flow fractionation of the samples revealed that the PCL-CNT had larger hydrodynamic diameters than PCL alone. Finally, the drug loading properties of the nanocomposites were assessed using docetaxel as the active substance. The nanocomposites showed comparable entrapment efficiencies of docetaxel (89%) to the CNTs alone (95%) and the PCL nanoparticles alone (81%). This is a facile method for obtaining non-spherical nanocomposites that combines the properties of PCL and CNTs such as the high aspect ratio, modulus. The high drug entrapment efficiency of these nanocomposites may have promising applications in drug delivery.

## Abstract 2

Facile route to polymer carbon nanotube nanocomposites.

## Graphical abstract

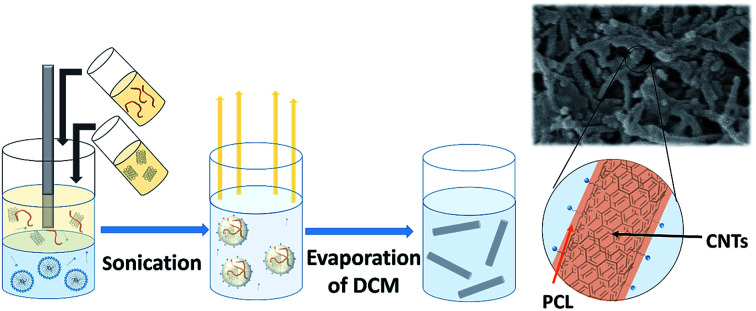

## Introduction

The use of nanomaterials for applications in drug delivery has been shown to offer a wide range of potential benefits such as encapsulating both hydrophilic and hydrophobic substances, improving the stability of drugs and providing targeted delivery.^1^ These benefits have been particularly well demonstrated in the treatment of cancer where a large number of therapies are now used clinically.^2,3^ However, effectively maximising the dose of the drug at the target site *versus* systemic distribution still remains a considerable challenge for any therapy. Recently, there has been growing interest in the influence of the geometry and stiffness of nanomaterials on their interaction with cells and tissues.^4–11^ A wide range of geometries and structures are synthetically possible such as nanoparticles,^12–14^ nanotubes,^15–17^ nanodisks,^18^ nanoshells^19,20^ and nanowires.^21^ The size, shape, stiffness and surface chemistry of nanomaterials has been shown to influence cytotoxicity,^22^ drug release,^23^ targeting and imaging contrast efficiency.^24^ Generally, high aspect ratio (length/diameter) nanomaterials can provide improved drug delivery potential in comparison to spherical nanoparticles.^4,5^

Carbon nanotubes (CNTs) are a type of high aspect ratio nanomaterial and they possess a range of interesting properties including their nanoscale size, unique fibre structure, large surface area and high mechanical stiffness.^15^ These properties have led to CNTs being investigated as drug delivery systems.^16,25^ CNTs have shown benefits in DNA delivery,^26^ use as cancer theranostics,^27,28^ small molecule drug delivery^16^ and regenerative medicine.^29^ CNTs can be covalently modified with drug molecules, or they can physically adsorb aromatic drugs *via* the strong π–π and hydrophobic interactions between the drug and the aromatic surface of the CNT. Such physical adsorption of drugs has been exploited for loading anthracyclines, a class of anticancer drug.^16^ However, in spite of their drug loading potential, the inherently hydrophobic nature of CNTs can limit their application in aqueous environments. As such, surface modification and coating is often utilised to improve the colloidal stability of CNTs.^30^ The cytotoxicity and biocompatibility of CNTs has been shown to be dependent on many factors such as the route of administration, the size and type of the CNTs (MWNT or SWNT) and presence of any surface modification;^31,32^ generally higher surface functionalisation of CNTs reduced cytotoxicity.^33^ Polymers are typically used for surface modification and these have been grafted to the surfaces of CNTs by amidation, radical coupling, esterification and other reactions.^34^ Alternatively, grafting from the surface of CNTs by anionic/cationic polymerizations or atom transfer radical polymerization has also been used.^35,36^ An attractive coating for CNTs would be poly(ε-caprolactone) (PCL), a polymer that has been widely investigated for drug delivery^12^ and a component of clinically approved devices.^37^ A number of composites of CNTs and PCL have been shown with the presence of the CNTs in a matrix of PCL, however, the majority of these are in the form of bulk polymers,^38–40^ electrospun fibres,^41,42^ films,^43–45^ or foams.^46^ These materials have shown promising properties for biological applications. Mattioli-Belmonte *et al.* showed that high level of CNTs incorporation (>60% weight) into a bulk PCL matrix caused cytotoxicity and no difference in cell viability was observed at lower CNT concentrations between PCL alone or the PCL-CNT composite.^47^ Moreover, other researchers have reported enhanced cell proliferation when CNTs were incorporated into PCL composites prepared by electrospinning.^42^ It is therefore clear that the biological response to PCL CNT composites depends on a number of factors including the concentration of CNTs and the morphology of the matrix material.

There are only a small number of examples of CNTs and PCL nanocomposites in the form of nanoparticles, in which individual CNTs are coated in PCL.^36,48^ Additionally, these nanoparticulate nanocomposites are prepared by chemical attachment of the polymer to the surface of the CNT and the resulting high-aspect ratio nanostructure were only dispersible in organic solvents. An alternative approach is to modify the CNTs by non-covalent functionalisation. This approach is particularly attractive due to the relative simplicity of process and the fact that it introduces fewer defects to the graphitic structure of the CNTs.^49^ A few examples of this approach have been shown, these include coating of the CNTs with poly(ethylene glycol)-phospholipid conjugates,^50^ forming a biopolymer coating using layer-by-layer assembly,^51^ or stabilising the surface with Pluronic F-127, a commercially available surfactant.^52^ The chemical structure of adsorbed polymer has been shown to influence the cytotoxicity and oxidative stress caused by the CNTs, and in the case, adverse biological effects were seen at a concentration of CNTs of 50 μg mL^−1^.^53^ One issue with non-covalent modification of CNTs is that the forces between the coating and the CNT are typically weak and desorption may occur especially *in vivo*.^54^ To overcome this issue, we are hypothesising that the use of the oil-in-water emulsion solvent evaporation method with CNTs will enable the preparation of CNTs with a physically attached coating of solid PCL that would not be easily desorbed. It is well reported that water-dispersible PCL nanoparticles can be prepared by the facile oil-in-water emulsion solvent evaporation method. In this process the polymer solution is emulsified and nanoparticles are formed upon evaporation of the solvent for the polymer.^55^ Additionally, PCL nanoparticles can provide entrapment efficiencies exceeding 80% and drug loadings of up to 20%.^56^ The potential of combining CNTs with their high modulus with the improved drug loading with PCL, a medically-proven polymer, may provide unique nanomaterials with promising biomedical applications.

**Fig. 1:**
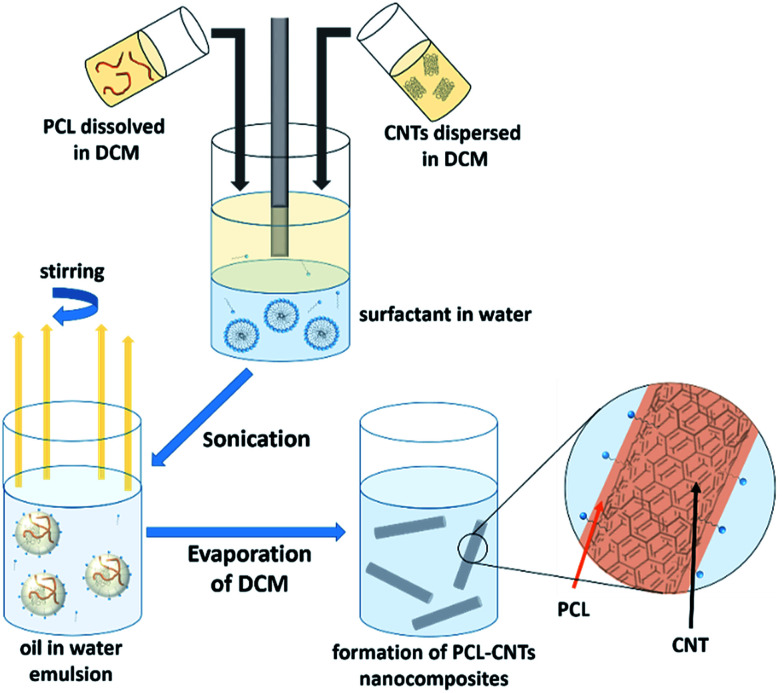
Scheme for the preparation of PCL-CNTs nanocomposites by an oil-in water emulsion solvent evaporation method. The solution of PCL and dispersion of CNTs (both in DCM) was emulsified in an aqueous surfactant solution. The resulting emulsion was kept stirring until the DCM had evaporated resulting in PCL-CNTs nanocomposites.

In this study, we demonstrate a facile approach to produce high-aspect ratio nanocomposites comprising of CNTs and PCL, with the aim of combining the benefits of both materials and encapsulating the anti-cancer drug docetaxel. We investigate the formation of nanocomposites by an oil-in-water emulsion solvent evaporation route (Fig. 1) and examine how to control the size and morphology of the particles. The nanocomposite composition and novel morphology of the particles are characterised by DLS, SEM, AFM, TGA and AF4. Finally, we investigate the potential for loading the nanocomposites with docetaxel and monitor the *in vitro* drug release.

## Experimental

### Materials

Polycaprolactone (PCL, molecular weight 14 000 g mol^−1^), sodium dodecyl sulphate (SDS), ammonium nitrate were purchased from Sigma-Aldrich. Non-functionalised multiwalled carbon nanotubes (MWCNTs) were purchased from Nanostructured & Amorphous Materials Inc. (Houston, USA) at 95% purity (Stock no. 1237YJS). According to the manufacturer, MWCNTs have an outside diameter 20–30 nm and length ranging between 0.5 and 2 μm. Docetaxel was purchased from Chemleader Biomedical. Dichloromethane (DCM) (analytical reagent grade), acetone, acetonitrile (HPLC grade) were purchased from AgraNova. Milli-Q water obtained from a water purification system had a resistivity of >18 MΩ cm^−1^ (PURELAB option R, Veolia). 10 kDa dialysis membrane was purchased from Spectrum Labs.

### Synthesis of PCL-CNTs

Nanocomposites of PCL and CNTs were prepared by an oil-in-water emulsion solvent evaporation method. CNTs were dispersed in 1 mL of DCM to form a dispersion with a concentration of 0.5 mg mL^−1^. The dispersion of CNTs was sonicated for 30 s using a Hielscher UP400 s ultrasonic processor (400 watts, 24 kHz, 45% of amplitude, 1 cycle). Next, the specific amount of PCL was dissolved in DCM (total volume 1 mL) to form the organic phase. The aqueous phase was prepared by dissolution of SDS in 4 mL of distilled water. The two organic phases were transferred into 14 mL glass vials containing the aqueous phase, dropwise through a needle (21G). In the next step, the immiscible phases were homogenised for 30 s using a Hielscher UP400s ultrasonic processor (400 watts, 24 kHz, 45% of amplitude, 1 cycle) to obtain an emulsion. The nanodispersion was mixed by magnetic stirring (500 rpm, hotplate model: Stuart US152D) overnight (∼16 h) to allow evaporation of DCM.

### Encapsulation of docetaxel into PCL-CNTs nanocomposites

PCL-CNTs nanocomposites with encapsulated docetaxel were prepared by an oil-in-water emulsion solvent evaporation method as previously described in the synthesis of PCL-CNTs method. CNTs were dispersed in 1 mL of DCM to form a dispersion with a concentration of 0.5 mg mL^−1^. Next, the specific amount of PCL was dissolved in DCM (total volume 1 mL) to form the organic phase. Docetaxel (DCX) was dissolved in 1 mL of acetone. The aqueous phase was prepared by dissolving SDS in 4 mL of distilled water. The three organic phases were transferred into 14 mL glass vials containing the water phase, dropwise through a needle (21G). In the next step, the immiscible solution was homogenised for 30 s using a Hielscher UP400s ultrasonic processor (45% of amplitude, 1 cycle) to obtain an emulsion. The nanosuspension was mixed by magnetic stirring (500 rpm) overnight ∼16 h to allow the DCM to evaporate. The same method was used to obtain PCL and CNTs alone.

### Characterisation

A Hitachi S-4800 cold Field emission (FE-SEM) scanning electron microscope (SEM) was used to image PCL NPs and PCL-CNTs. The samples to be analysed were centrifuged at 9000 rpm for 1 hours and washed with deionised water three times prior to addition (concentration 1 mg mL^−1^) to glass coverslips stuck onto an aluminium stubs using carbon double-sided sticky tabs from Agar Scientific, Essex, UK and left to evaporate overnight. The prepared samples were coated with gold for 2.5 minutes at 20 mA using an EMITECH K550X Sputter Coater.

Asymmetric flow field flow fractionation experiments were performed on an MT2000 with RI and UV-vis detectors from Postnova Analytics, Landsberg/Germany. The sizes of the samples were obtained by dynamic light scattering (DLS) using a Malvern Zetasizer Nano ZS (running Malvern Zetasizer software V7.11) (Malvern Instruments, Malvern, UK) with 633 nm He–Ne laser and the detector positioned at 173°, coupled online to the MT2000. A 350 μm spacer and 10 kDa regenerative cellulose (RC) membrane were installed in the separation channel. The conditions used for the separations was based on a method existing in the literature.^57^ Briefly, The mobile phase was 1 × 10^−5^ mol L^−1^ NH_4_NO_3_ in Milli-Q H_2_O. Type I distilled water was obtained from a water purification system had a resistivity of >18 MΩ cm^−1^ (PURELAB option R, Veolia). The injected volume was 30 μL of 1 mg mL^−1^ sample. The injection/focussing time was 5 min using a cross flow of 2 mL min^−1^. The cross flow rate was 2 mL min^−1^ for the first 15 min (*t*_0_–*t*_15_) in constant manner, and thereafter, the cross flow was linearly decreased from its initial value to 0 over a period of 5 min. Following the complete reduction in cross flow, the tip-flow continued for an additional 35 min. The UV-vis detector measured wavelength were monitored for 300 nm. The *Z*-average diameter and count rate were measured by an inline Malvern Zetasizer ZS DLS at 3 second intervals. DLS calculates the *Z*-average size of particles using the Stokes–Einstein equation.

AFM measurements of the samples were performed with the samples deposited on glass coverslips which were adhered to mica substrates. The samples were prepared on the glass coverslip stuck onto mica substrates. A few microliters of suspended sample (concentration ∼1 mg mL^−1^) were pipetted onto the mica surface and left to dry by exposing to air overnight (∼16 h). AFM imaging was conducted using a Bruker Multimode 8 instrument (Bruker, Santa Barbara, USA), operated in ambient conditions with a Bruker RTESPA-525 probe using the Peakforce Quantitative Nanomechanical Mapping (PFQNM) method.^58,59^ The RTESPA-525 probe has a nominal spring constant of 200 N m^−1^ and a tip radius of 8 nm. All scans were conducted at a scan rate of 0.576 Hz with a scan size of 2.00 μm.^58^

Thermo-gravimetric analyses (TGA) were performed in a simultaneous thermal analyser (STA) 449F1 Jupiter (Netzsch GmbH), which includes a thermo-gravimetric analyser. Samples of unprocessed CNTs, PCL nanoparticles and the PCL-CNT nanocomposites (5–15 mg) were added to platinum pan. In the case of the PCL nanoparticles and the PCL-CNT nanocomposites, these dispersions were prepared using the usual method and were then freeze-dried prior to addition of the dry powder to the analysis pans. The atmosphere of the samples, the pan and the sample holder were evacuated and purged with argon three times to remove the air before analysis. The samples were then analysed by heating at a heating rate of 10 °C min^−1^ to 700 °C in an atmosphere of argon flowing at 20 mL min^−1^, whilst monitoring weight changes at a resolution of ±25 ng.

### Quantification of docetaxel by HPLC

HPLC measurements were performed with the use of a PerkinElmer Series 200 instrument. The chromatographic conditions were used as previously described in the literature.^60^ The chromatograph column used was an Agilent Zorbax Eclipse Plus C18. Solvent A contained HPLC grade water and solvent B consisted of HPLC grade acetonitrile. The flow rate of the mobile phase was 1.0 mL min^−1^. The HPLC gradient was kept as *T*/% *B* (*T* is time and *B* is a percentage of acetonitrile solvent): 0/35, 15/65, 25/75, 30/95, 35/100, 39/100 and 40/35 with a post run time of 5 min. The column was maintained at 25 °C. The detection wavelength was 230 nm. The injection volume was 10 μL. The diluent used was a 1 : 1 mixture of water and acetonitrile.

A calibration curve was prepared for docetaxel from 14 standard solutions. Samples were prepared by dissolving the appropriate amount of docetaxel in 1 : 1 mixture water and acetonitrile. A linear calibration plot for the above method was obtained over 3.9 μg mL^−1^ to 250 μg mL^−1^. The correlation coefficient was 0.99.

In order to calculate the encapsulation efficiency of the samples the amount of ‘free’ docetaxel (*i.e.* not encapsulated in the nanoparticles) was measured. Firstly, the samples loaded with docetaxel were freeze dried for 24 h to remove the water. After that, the freeze dried samples were dispersed in 2 mL of HPLC methanol for 15 min. The methanol was then transferred to a spin filter tube (cut off 3.5 kDa) and centrifuged at 6000 g for 1 h at 20 °C. The filtered solution was then analysed by HPLC using the same method and column as for the drug release study.

### *In vitro* drug release

Briefly, 4 mL of PCL, CNTs and PCL-CNTs-PLGA nanoparticles solution (1 mg mL^−1^) were introduced into dialysis membrane bag (12–14 kDa, Spectrum Laboratories Inc.) The end-sealed dialysis bag was incubated into 500 mL of distilled water in the 25 °C. Every 24 h, 250 mL of water was taken and freeze dried to remove water. After that, the freeze dried samples were dispersed in 2 mL of 1 : 1 mixture water and acetonitrile. Solution was analysed by HPLC, using the same method as described in HPLC method section. The release media was changed every 24 h.

## Results and discussion

### Influence of the concentration of SDS, CNTs and PCL on particle size and morphology

**Fig. 2:**
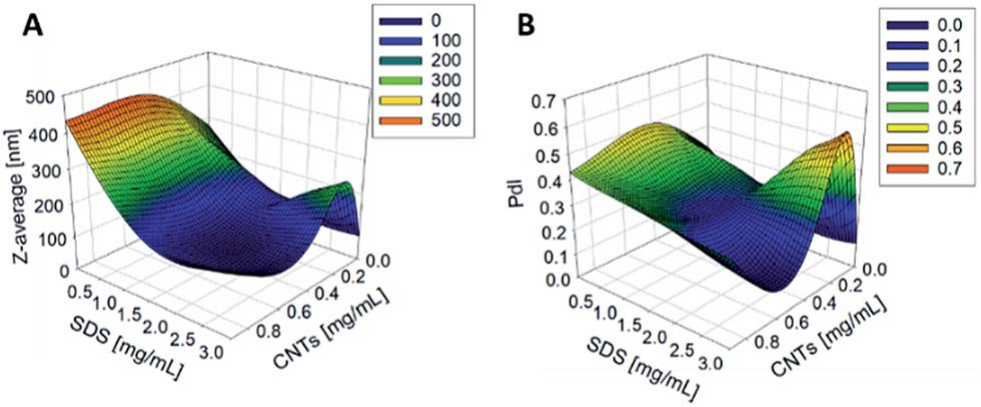
The effect of changing the concentration of CNTs and SDS on the *Z*-average diameter (A) and the PdI (B) of the resulting nanoparticles as measured by DLS. The concentration of PCL was 6 mg mL^−1^.

The synthesis of PCL-CNTs nanocomposites was attempted by an oil-in water emulsion solvent evaporation method (Fig. 1), PCL and CNTs were contained in DCM which was then emulsified by sonication with an aqueous phase of the surfactant sodium dodecyl sulphate (SDS). The DCM was then left to evaporate under ambient conditions. Two variables were initially investigated: the concentration of SDS in the aqueous phase and the concentration of CNTs in the oil phase, with the concentration of PCL kept constant at 6 mg mL^−1^. We observed that samples containing higher concentrations of CNTs produced a turbid emulsion which was a darker grey-black in colour. The samples were then purified by centrifugation to remove any excess surfactant before being analysed by DLS. The analysis of the samples by DLS provided the mean hydrodynamic diameters of the particles and a measure of the broadness of the particle size distribution, quantified by the polydispersity index (PdI) (Fig. 2). It was found that particles without SDS had the largest diameters and that there was a further increase in the size as the CNT concentration increased from 0.03 mg mL^−1^ to 0.9 mg mL^−1^ of CNTs, with a diameter ranging from 289 to 434 nm respectively (Fig. 2A). The same effect was visible for PdI results; the absence of surfactant resulted in the highest PdI values (Fig. 2B). Without SDS, the high interfacial tension between water and DCM would result in an emulsion with low stability, this will have led to droplet coalescence upon evaporation of the DCM, giving a higher mean particle diameter and broad particle size distribution. An SDS concentration of 1.5 mg mL^−1^ produced the smallest particles and the lowest PdIs for all of the three CNT concentrations. Concentrations above 1.5 mg mL^−1^ of SDS did not reduce particle size further suggesting 1.5 mg mL^−1^ was sufficient concentration of surfactant to minimise droplet coalescence, further increases in concentration generally increased the mean diameter or polydispersity index (Fig. 2). At the lowest concentration of SDS tested (below 0.5 mg mL^−1^) and a high concentration of CNTs (above 0.6 mg mL^−1^) particles were obtained with larger mean diameters. The presence of the CNTs which had a mean length of 425 nm (see (ESI), Fig. S1†) in the DCM may have resulted in non-spherical droplets with a larger apparent hydrodynamic diameter. DLS analysis generally showed monomodal distributions for the samples (See ESI, Fig. S2†) with the PdI ranging between 0.169 (6 mg mL^−1^ PCL 0.03 mg mL^−1^ CNTs and 1.5 mg mL^−1^ SDS) and 0.428, a multimodal distribution (6 mg mL^−1^ PCL, 0.9 mg mL^−1^ CNTs and 0 mg mL^−1^ SDS). The concentration of SDS significantly impacted the results. When there was SDS no present broad size distributions were obtained, whereas when the SDS concentration was above 1.5 mg mL^−1^ smaller particles and a narrower distributions was obtained.

**Fig. 3:**
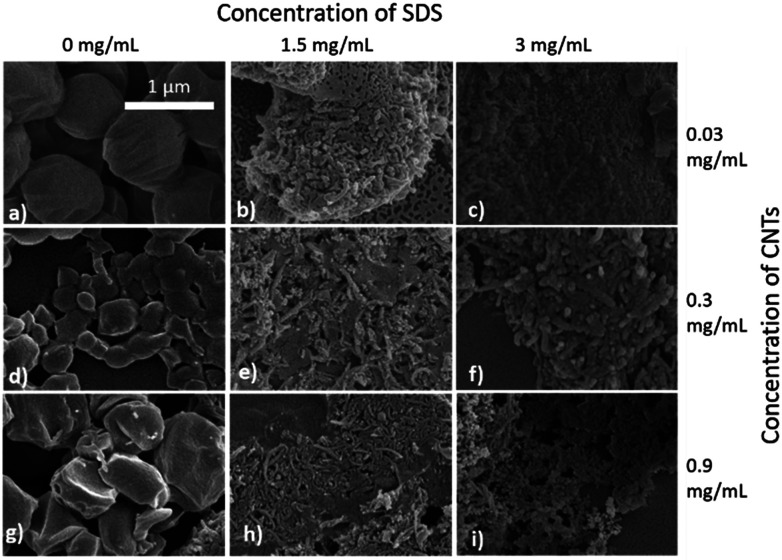
SEM images of the nanomaterials showing the influence of SDS and CNT concentration on the different sizes and morphologies of the nanocomposites of PCL/CNTs. (a) 6 mg mL^−1^ of PCL, 0.03 mg mL^−1^ of CNTs and 0 mg mL^−1^ SDS (b) 6 mg mL^−1^ of PCL, 0.03 mg mL^−1^ of CNTs and 1.5 mg mL^−1^ of SDS (c) 6 mg mL^−1^ of PCL, 0.03 mg mL^−1^ of CNTs and 3 mg mL^−1^ of SDS (d) 6 mg mL^−1^ of PCL, 0.3 mg mL^−1^ of CNTs and 0 mg mL^−1^ of SDS (e) 6 mg mL^−1^ of PCL, 0.3 mg mL^−1^ of CNTs and 1.5 mg mL^−1^ of SDS (f) 6 mg mL^−1^ of PCL, 0.3 mg mL^−1^ CNTs and 3 mg mL^−1^ of SDS (g) 6 mg mL^−1^ of PCL, 0.9 mg mL^−1^ of CNTs and 0 mg mL^−1^ of SDS (h) 6 mg mL^−1^ of PCL, 0.9 mg mL^−1^ of CNTs and 1.5 mg mL^−1^ of SDS (i) 6 mg mL^−1^ of PCL, 0.9 mg mL^−1^ of CNTs and 3 mg mL^−1^ of SDS. All samples were dispersed in 4 mL of distilled water. Scale bar applies to all images.

**Fig. 4:**
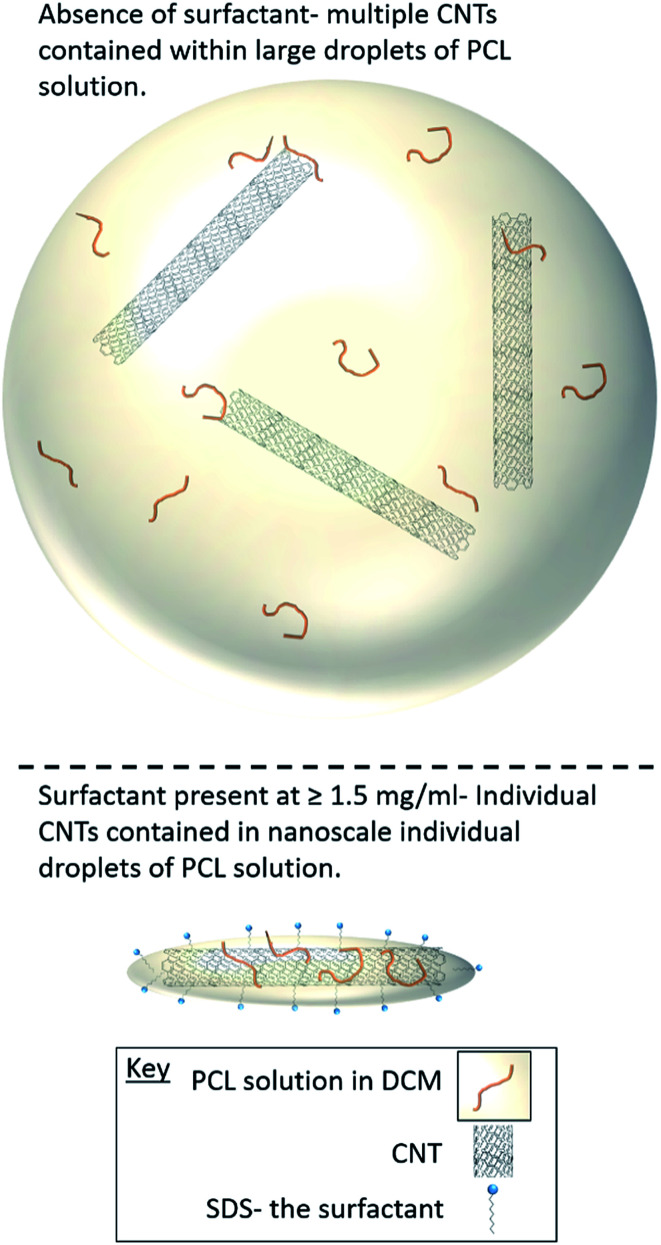
Cartoon representation of how the surfactant concentration during the emulsification process influences the morphology of the nanocomposites.

Selected samples were then further characterised by scanning electron microscopy (SEM) to provide information on the morphology and particle size in the dried state, as shown in Fig. 3. In order to minimise charging during imaging the samples were sputter-coated with gold. All the samples appeared to have agglomerated upon drying. The length and width of the particles was then determined from the SEM images. When no SDS was used large spherical particles (392–659 nm depending on CNTs concentration) were obtained with diameters approximately matching those determined by DLS. As previously mentioned, it is likely that in the absence of surfactant the interfacial tension between the water and DCM resulted in droplet coalescence. When the concentration of the CNTs was increased it resulted in particles with more irregular, less spherical morphology. At higher concentrations of SDS (1.5 mg mL^−1^ and 3 mg mL^−1^) and with CNTs present, rod-like morphologies were observed for all samples, these structures closely resemble those previously reported in the literature for PCL-CNT nanocomposites prepared by a covalent modification with either a “grafting from” approach^36^ or click chemistry method.^48^ This behaviour was likely due to the SDS lowering the interfacial tension between the DCM and the aqueous continuous phase resulting in smaller emulsion drops. The CNTs will preferentially be wet by DCM rather than water due to the hydrophobic nature of CNTs.^61^ As the diameter of a DCM droplet approaches the length of the CNT dispersed within the droplet there is the potential for the CNT to be exposed to the surrounding aqueous continuous phase as the DCM evaporates. Minimisation of the interfacial energy of the system will lead to the DCM–water interface growing rather than create new CNT–water interface. This behaviour would likely result in a non-spherical droplet being formed. It can then be expected that as the DCM continued to evaporate the PCL would be deposited onto the surface of the CNT. These experiments suggest that the presence of the surfactant would have allowed smaller droplets of DCM containing the PCL and CNTs to form, upon evaporation of the DCM non-spherical nanomaterials were then formed (see Fig. 4 for a visual representation of this hypothesis).

The PCL-CNT nanocomposites displayed colloidal stability in water for at least 5 days with very little change in the mean diameter and polydispersity over that time period (see ESI, Fig. S3†). In phosphate buffered saline, a common mimic for physiological fluids, the nanocomposites showed colloidal instability as the ions in the salt screened out the electrostatic stabilisation of the particles (see ESI, Fig. S4†). We believe that this issue can be address in the future by the use of a polymer stabiliser that will provide steric stabilisation to the nanocomposites. The reproducibility of synthesis of the PCL-CNT samples was also tested, three selected samples were reproduced, in these the concentration of PCL used 3 mg mL^−1^ and with differing concentrations of CNTs (0.01–0.3 mg mL^−1^) and SDS (0–1 mg mL^−1^). In each case the standard deviations of the replicates were found to be less than 10% of the mean diameter of the sample (see ESI, Fig. S5†). This finding showed that the oil-in water emulsion solvent evaporation method is a robust and reliable process for obtaining non-spherical nanomaterials. From the range of concentrations that were tested, a single formulation was selected for each type of nanodispersion: PCL alone nanoparticles, a CNT alone dispersion and a PCL-CNT nanocomposite (see Table 1). These samples were then prepared for further analysis and comparison between the properties of the different nanostructures.

**Table 1:** Summary of the concentrations and compositions of the samples of PCL nanoparticles, CNT dispersion and PCL-CNT nanocomposites prepared for further investigation

Sample	Concentrations used in sample preparation (mg mL^−1^)			Composition (%)	
	PCL	CNTs	SDS	PCL	CNTs
PCL	10	0	1.5	100	0
CNTs	0	0.9	1.5	0	100
PCL-CNTs	10	0.9	1.5	93	7

### The morphology and properties of the sample as studied by AFM SEM and TGA

**Fig. 5:**
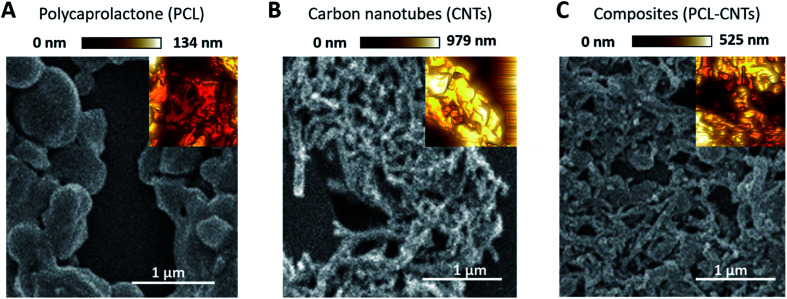
Analysis of the PCL, CNT and PCL-CNT (93% PCL, 7% CNTs by mass) nanocomposites by SEM and AFM (inset): (A) PCL alone, (B) CNTs and (C) PCL-CNTs composites. The AFM images are based on the peak force error measurements. The AFM images were not obtained from the same region shown in the SEM image, but have been inset so as to assist direct comparison with the morphologies revealed both techniques at the same scale. The scale bar applies to both the SEM images and inset AFM images.

SEM and AFM characterisation was then conducted to further investigate the surface, morphology and mechanical properties of the PCL-CNT nanocomposite; this was compared to PCL nanoparticles and CNTs alone (Fig. 5). The same regions of each sample was imaged by both SEM and AFM, however, it was not possible to image precisely the same location with both techniques. In the preparation of these samples no gold coating was used for the SEM as this would invalidate the mechanical characterisation of the sample by AFM analysis. Therefore, in order to prevent surface charging the SEM was operated in deceleration mode, which led to a slight reduction of the image quality. As before, the sample of PCL alone consisted of spherical particles with a mean diameter of 382 nm and appeared to agglomerate upon drying (Fig. 5A). Analysis of the sample of CNTs alone revealed fibres with varying lengths (200–795 nm with a mean length 324 nm and width 53 nm) (Fig. 5B), a similar morphology was observed for the nanocomposite sample containing both PCL and CNT (Fig. 5C) where the sample consisted of high aspect ratio fibres with a mean width of 55 nm and mean length of 172 nm (see ESI, Fig. S6–S9† for the images showing where the measurements made for each samples). Due to the agglomeration upon drying, the length measurements should be interpreted with caution because it was often unclear where one non-spherical nanoparticle ended, however, the larger widths of the PCL-CNTs nanocomposites compared to the CNTs alone suggested that the PCL has deposited on the surface of the CNTs. The SEM characterisation data provides strong evidence that the process of combining the PCL and CNTs in the same phase during the oil-in water emulsion solvent evaporation method results in nanocomposite rods that consist of CNTs coated in PCL.

**Fig. 6:**
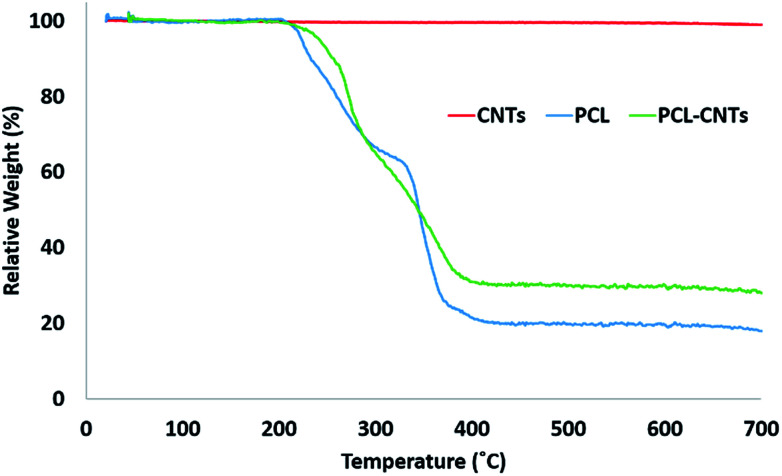
Analysis of the PCL-CNT (93% PCL, 7% CNTs by mass) nanocomposite and its constituents by TGA. The concentrations used during preparation were 10 mg mL^−1^ of PCL and 1.5 mg mL^−1^ SDS for PCL; 10 mg mL^−1^ of PCL, 1.5 mg mL^−1^ SDS and 0.9 mg mL^−1^ CNTs for PCL-CNTs. CNTs consists of 0.9 mg mL^−1^ CNTs alone.

In order to provide more evidence for the preparation of PCL/CNT nanocomposites AFM was utilised to characterise the samples and measure the elastic modulus of all three samples (AFM images are shown as insets in Fig. 5). Literature values for the elastic modulus of PCL and CNT are 600 MPa^62^ and 1 TPa^63^ respectively. AFM nanomechanical analysis measures the properties associated with 2 nm depth surface of material,^59^ therefore a modulus for approximately 600 MPa was expected in the event of successful PCL-CNT nanocomposites. The Peakforce quantitative nanoscale mechanical method relies on the selection of an appropriate stiffness cantilever depending on the expected elastic modulus of the sample. However, no probe of an appropriate stiffness was available for the CNTs. Hence, we conducted all the AFM experiments using the same probe. The probe used in this experiment (TAP525) is recommended by the manufacturer (Bruker) for samples with elastic modulus values in the range 1–20 GPa. Therefore, it was not possible to obtain reliable modulus values for the CNTs alone. However, analysis of the DMT^64^ modulus for both the PCL and PCL-CNT nanocomposite provide comparatively similar values of 770 and 560 MPa respectively, indicating that all the CNTs have been coated with at least 2 nm of PCL. The moduli of both samples were consistent across the surfaces, and no regions of very high modulus were observed in the nanocomposite sample which would have indicated uncoated CNTs (see ESI, Fig. S10†). While the nanocomposites showed a similar surface modulus to that of PCL, it is likely that high modulus of the CNT would mean that the bulk modulus of the nanocomposites would be comparable, this could be of particular interest for drug delivery applications as it has previously been shown that materials with higher moduli display greater cellular uptake.^65^ The fibrous morphology observed for the PCL-CNT sample by SEM and the stiffness as determined by AFM further supports the concept that the CNTs have been coated with PCL to give high aspect ratio nanocomposites. Thermo-gravimetric analysis (TGA) was also used to provide information on the thermal stability and CNT content of the PCL-CNT nanocomposites.^36,41,66^ The decomposition behaviour of the CNTs and PCL nanoparticles and PCL-CNT nanocomposites are shown in Fig. 6. The CNTs were found to show thermal stability over the temperature range tested, in agreement with the literature.^67^ Literature data on SDS alone has previously shown to display a mass loss commencing at 200 °C until 300 °C at which 73% of the mass of the compound has been lost,^67^ while PCL has been shown to degrade when heated above 360 °C under inert atmosphere with no residue mass remaining at 500 °C.^68^ In the context of this information, the start of degradation for the sample of PCL nanoparticles stabilised by SDS began at approximately 210 °C which was likely due to the degradation of the SDS up to approximately 300 °C where the data exhibits a shoulder. This TGA curve steepens abruptly at *ca.* 345 °C that is likely associated with the start of the degradation of the PCL. Above 400 °C very little further mass loss was noted and the residue mass of 19.6% at 600 °C was due to the SDS. For the PCL-CNT nanocomposite sample, the onset of the mass loss was approximately 220 °C which also corresponded to the degradation SDS within the nanocomposite. The increase in the degradation temperature of the SDS could potentially suggest that the formation of the nanocomposite structure has increased the thermal stability of the SDS in the nanocomposite. A slight shallowing of the TGA curve was observed at 290 °C which may be associated with completion of the SDS degradation. The curves steepens again around 345 °C, which may be associated with the PCL beginning to degrade. This effect was less pronounced in the PCL-CNT nanocomposite compared to the PCL nanoparticles which may be due to the presence of the CNT in the nanocomposite altering the degradation behaviour of the PCL.^48^ Above 400 °C no further mass loss was seen for the nanocomposite and the residue mass at 600 °C was 29.2%. The PCL-CNT nanocomposite showed a higher residue mass compared to the PCL alone nanoparticles. This 9.6% of the mass can be attributed to the CNTs present within PCL-CNT nanocomposites, which matches a literature value of 10% for PCL grafted CNTs.^48^ This mass is higher than expected (the theoretical composition of the sample was 93% PCL, 7% CNTs by mass) and may be attributed to a slight increase in the concentration of the CNT dispersion due to some evaporative loss of DCM during sonication prior to the emulsification. Raman measurements were also used to characterise PCL, CNTs and PCL-CNTs particles. The presence of the peaks for both the PCL and CNT indicates that the nanocomposite sample contains both of PCL and CNT (see ESI, Fig. S11†).

### Fractionation of the particles by asymmetric flow field flow fractionation and measurements of hydrodynamic diameters

**Fig. 7:**
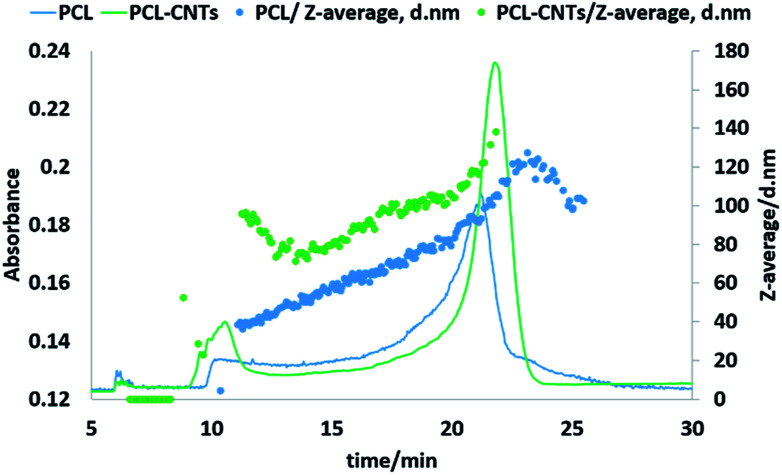
Fractogram of PCL and PCL-CNTs (93% PCL, 7% CNTs by mass) obtained from asymmetric flow field flow fractionation coupled online with UV-VIS detector. Hydrodynamic diameters were obtained from DLS coupled online.

Next, the PCL alone and the PCL-CNT nanocomposite samples were further analysed by asymmetric flow field flow fractionation with the aim of obtaining high resolution sizing of the nanoparticles in the dispersed state. High resolution sizing would make it possible to determine if the PCL/CNT nanocomposites consisted of a single population of particle rather than resulted in two separate populations of PCL or CNTs alone. Asymmetric flow field flow fractionation separates samples based on hydrodynamic diameter^69^ with smaller particles eluting before larger particles, the fractionated sample then passes through inline detectors to determine the diameter of the particles. The separation conditions were chosen from previous studies of CNTs.^57^Fig. 7 shows the data for the asymmetric flow field flow fractionation analysis of the samples combining the absorbance from UV-VIS detector and *Z*-average diameter as measured by inline DLS. The UV-vis detector was set to a wavelength of 300 nm, chosen based on absorbance spectra of PCL and CNTs, and thus provides information on the concentration of the particles. The samples showed good separation and the absence of a void peak revealed that aggregation has not occurred in the system. The sample that consisted of PCL alone was found to consist of a higher dynamic diameter distribution between 11 nm and 115 nm, with the mode of the UV-vis measurement equating to a diameter of 93 nm. The size range of PCL-CNTs sample was between 11 nm and 350 nm with the mode of the UV-vis measurement equating to a diameter of 138 nm. A sample of CNT alone was also analysed but is not directly comparable to the PCL and nanocomposites, the uncoated CNT surface will likely have a different interaction with the membrane in the AF4 compared to the other samples with the PCL coating which may have resulted in different elution behaviour. The CNTs alone consisted of a distribution of 16 to 187 nm (see ESI, Fig. S12†) The PCL-CNT nanocomposites were found to have larger hydrodynamic diameters compared with pristine CNTs and PCL alone. The measurements obtained from the detector coupled online with asymmetric flow field flow fractionator showed higher intensity for PCL-CNTs compared to the PCL and CNT, which was due to the higher concentration of composites in the sample.

As previously stated, asymmetric flow field flow fractionation separates a sample based on the hydrodynamic diameter; particles of the same diameter should elute at the same time. However, as shown in Fig. 7, PCL or PCL-CNT nanocomposite particles with the same hydrodynamic diameter eluted at considerably different times. The PCL-CNT nanocomposites tended to elute before the PCL alone particles. Similar behaviour for non-spherical particles has been published by Gigault *et al.* in which they showed that longer gold nanorods (GNRs) with the same diameters eluted earlier than shorter GNRs.^70^ It has been suggested that elution of non-spherical particles depends on an aspect ratio and steric-entropy contribution associated with their orientation. Gigault *et al.* suggested that GNRs located higher in the channel elute faster than associated closer to the membrane.^70^ We believe that the earlier elution of the PCL-CNT nanocomposites compared to the spherical PCL alone nanoparticles was due to their higher aspect ratio of the nanocomposites.

### Drug loading and release

Finally, the drug loading and release from PCL, CNTs and PCL-CNTs nanocomposites was investigated. In this work docetaxel, an effective anticancer drug^71–73^ was used. Docetaxel (DCX) has some clinical limitations associated with its poor water solubility.^74^ The anticancer drug was encapsulated in the nanocomposite carriers, while PCL alone and CNTs alone were also tested as controls. Drug encapsulation was achieved by including DCX into the oil phase for the particle preparation. The resulting DCX loaded samples were analysed by DLS (as an unfractionated sample) to provide a mean diameter, PdI and distribution plot. Table 2 shows the DLS results for PCL, CNT and PCL-CNTs. The nanocomposites showed larger mean diameters compared with PCL and pristine CNT. The DLS distribution graph for the PCL alone sample was monomodal. (See ESI, Fig. S13†). The CNTs and PCL-CNTs have a PdI of 0.33 which means that the distribution is broader than the PCL alone particles, this is likely due to the polydisperse nature of the CNTs themselves which gave a PdI value of 0.33. Hydrodynamic diameter and PdI of the three samples were also measured after encapsulation of DCX. The size and PdI of CNTs alone were higher compared with unloaded CNTs alone. These results potentially indicate that molecules of anti-cancer drug adsorbed to the surface of the CNTs may have possibly resulted in some aggregation of the CNTs. The properties of CNTs have previously shown that loading *via* π–π stacking is possible for DCX.^75^ It was found that after encapsulation, PCL and PCL-CNTs showed a lower diameter and PdI. In this case it is possible another type of interaction between the PCL/CNTs and DCX may have altered the size of the PCL–DCX nanoparticles and PCL-CNTs-DCX nanocomposites. The entrapment efficiency (EE) was also analysed by HPLC (EE_CNT_ = 95%, EE_PCL_ = 81% and EE_PCL-CNT_ = 89%).

**Table 2:** Summary of the diameter and PdI for samples without and with encapsulated DCX obtained from DLS

	Unloaded		Loaded DCX	
	*Z*-average/diameter nm	PdI	*Z*-average/diameter nm	PdI
PCL	90 ± 1	0.160 ± 0.008	73 ± 6	0.180 ± 0.009
CNTs	149 ± 7	0.330 ± 0.007	314 ± 10	0.530 ± 0.034
PCL-CNTs	215 ± 8	0.330 ± 0.013	162 ± 5	0.400 ± 0.019

**Fig. 8:**
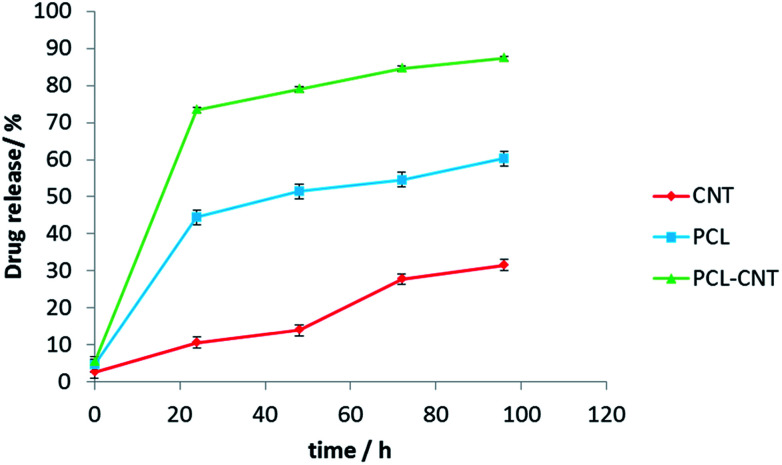
The graph presents docetaxel releasing study (% of drug released *vs.* time) for CNTs, PCL and PCL-CNTs (93% PCL, 7% CNTs by mass) obtained from HPLC analysis. The data showed three different profiles for releasing anti-cancer drug.

*In vitro* release experiments were then conducted to observe the drug release behaviour of the nanocomposites. The drug released over time is presented in Fig. 8. The nanocomposites and CNT alone encapsulated more anti-cancer drug compared with PCL alone. The release of DCX from different systems has been studied previously.^76,77^ In those studies, the authors showed that pH, temperature, solvent and type of carrier have an effect on drug release. Our release data showed that DCX is released most rapidly from the PCL-CNTs. The slowest profile was found with the CNTs alone with 31% of the drug released in 96 hours matching the DCX release behaviour from CNTs previously shown in the literature.^78,79^ The difference in the release profiles between the CNTs and the PCL-CNT nanocomposites may be due to the different structures of the nanocarriers. In the case of the PCL-CNT nanocomposites, the PCL coating the surface may have disrupted the ability for the DCX to adsorb onto the CNT. This is supported by the literature in which it is stated that drug release behaviours from CNTs are altered when different polymer surface functionalisations are present.^78^ No degradation of the drug was detected after the drug release from all of the samples (examples of the chromatograms obtained from HPLC analysis are shown in the ESI, Fig. S14–S17†). These findings show that not only can the PCL-CNT successfully encapsulate DCX but also has the ability to release it. More interestingly, the difference in the release behaviour of DCX from the different samples suggests that there is a potential to tune the release profile by varying the composition of the nanocomposites.

## Conclusions

In the present study, PCL-CNT nanocomposites were prepared by a facile oil-in water emulsion solvent evaporation method. This approach produced rod-like, non-spherical nanoparticles. Different sizes and morphologies of nanoparticles were produced depending on the concentration of SDS, CNTs and PCL. The samples with rod-like morphologies and the lowest diameter and PdI were synthesised using 1.5 mg mL^−1^ of SDS, 0.9 mg mL^−1^ of CNTs and 10 mg mL^−1^ PCL. The AFM analysis revealed that the incorporation of the CNTs in the nanocomposites did not increase the modulus of the particles, including the successful surface coverage of the CNTs with PCL. In addition, TGA analysis of the PCL-CNT nanocomposite also showed that they contained 9.6% CNTs by mass. Asymmetric flow field flow fractionation measurements showed good separation of particles and the PCL-CNTs had higher hydrodynamic diameters than PCL and CNTs alone. Raman data indicated the presence of both PCL and CNTs in the nanocomposites. DCX loading in PCL, CNTs and PCL-CNTs were determined by HPLC and showed high entrapment efficiencies (CNT = 95%, PCL = 81% and PCL-CNT = 89%). Moreover, faster release of DCX from PCL-CNTs was observed with about 90% of the drug released from the nanocarriers after approximately 100 h.

To the best of our knowledge this is the first example of producing high-aspect ratio PCL-CNT nanocomposites using a simple oil-in water emulsion solvent evaporation method. The high entrapment efficiencies of the nanocomposites offer the potential for these nanocomposites to be used in drug delivery applications. We hypothesise that the needle-like morphology and high modulus of the nanocomposites will enhance the cellular uptake of anticancer drugs and the coating with PCL will potentially reduce any cytotoxicity associated with the pristine CNTs.

## Conflicts of interest

There are no conflicts to declare.

## Supplementary Material

RA-008-C7RA13553J-s001
