# Supplementary material for: Facile production of nanocomposites of carbon nanotubes and polycaprolactone with high aspect ratios with potential applications in drug delivery
Source: RSC Adv. 2018 May 4;8(30):16444–54. doi: 10.1039/c7ra13553j (PMC6003547; doi:10.1039/c7ra13553j)
Supplement: RA-008-C7RA13553J-s001 [file RA-008-C7RA13553J-s001.pdf]

Electronic Supplementary Material (ESI) for RSC Advances.
This journal is © The Royal Society of Chemistry 2018

Supporting Information

Facile Production of Nanocomposites of Carbon Nanotubes and Polycaprolactone with High Aspect Ratios with Potential Applications in Drug Delivery

Edyta Niezabitowska,[a] Jessica Smith,[a] Mark R. Prestly,[a] Riaz Akhtar,[b] Felix W. von Aulock,[c] Yan Lavallée,[c] Hanene Ali-Boucetta[d] and Tom O. McDonald

Addresses:

a) Department of Chemistry, University of Liverpool, Crown Street, Liverpool, L69 7ZD, United Kingdom.
b) Biomechanical Engineering Group, School of Engineering, University of Liverpool, Brownlow Hill, Liverpool, L69 3GH, United Kingdom.
c) Institute of Clinical Sciences, College of Medical and Dental Sciences, University of Birmingham, Edgbaston, Birmingham, B15 2TT, United Kingdom.

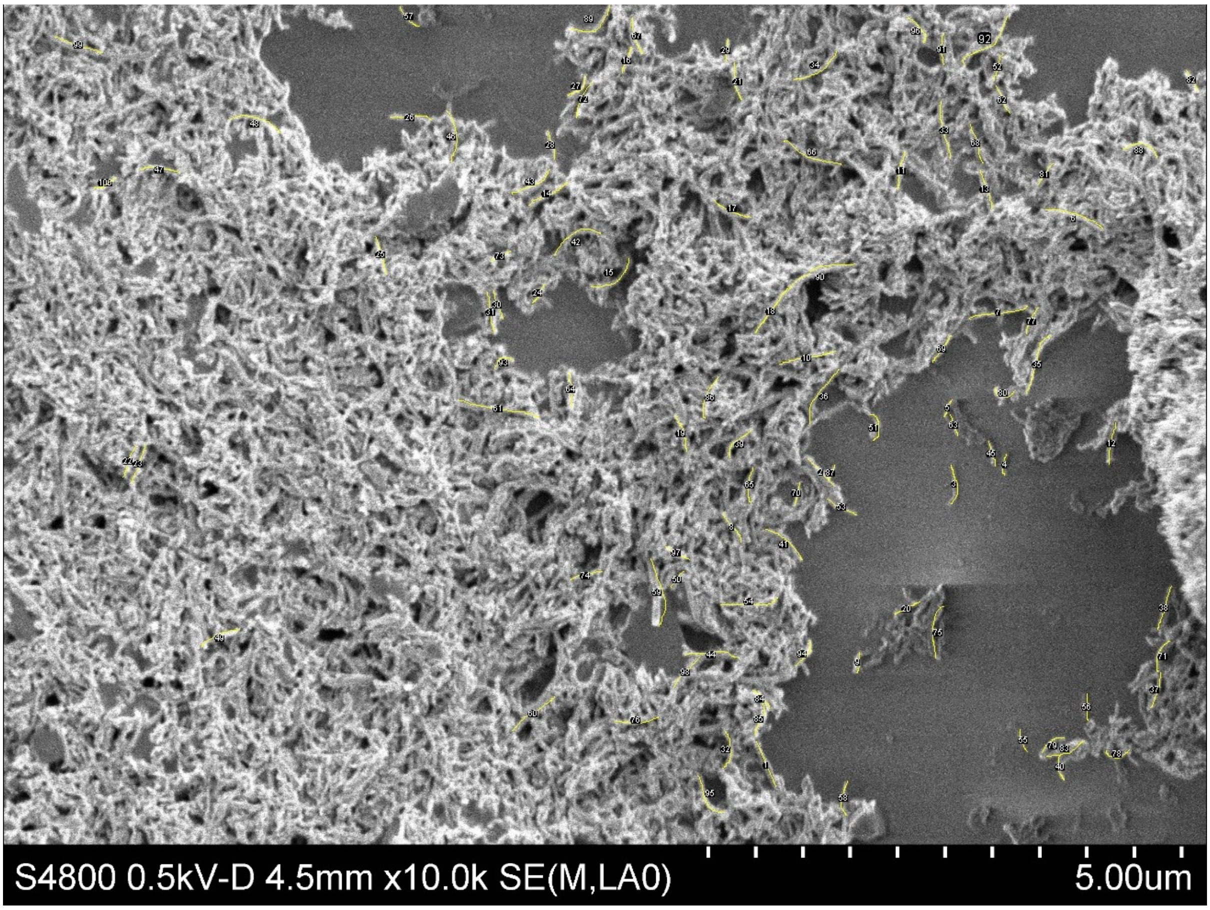

Figure S1. SEM of CNTs alone dried onto a glass substrate. The yellow lines indicates the measurement of the length CNTs particles obtained by ImageJ. The mean length of the CNTs was determined to be 425 nm (standard deviation 145 nm) and the mean width was 60 nm (standard deviation 14 nm).

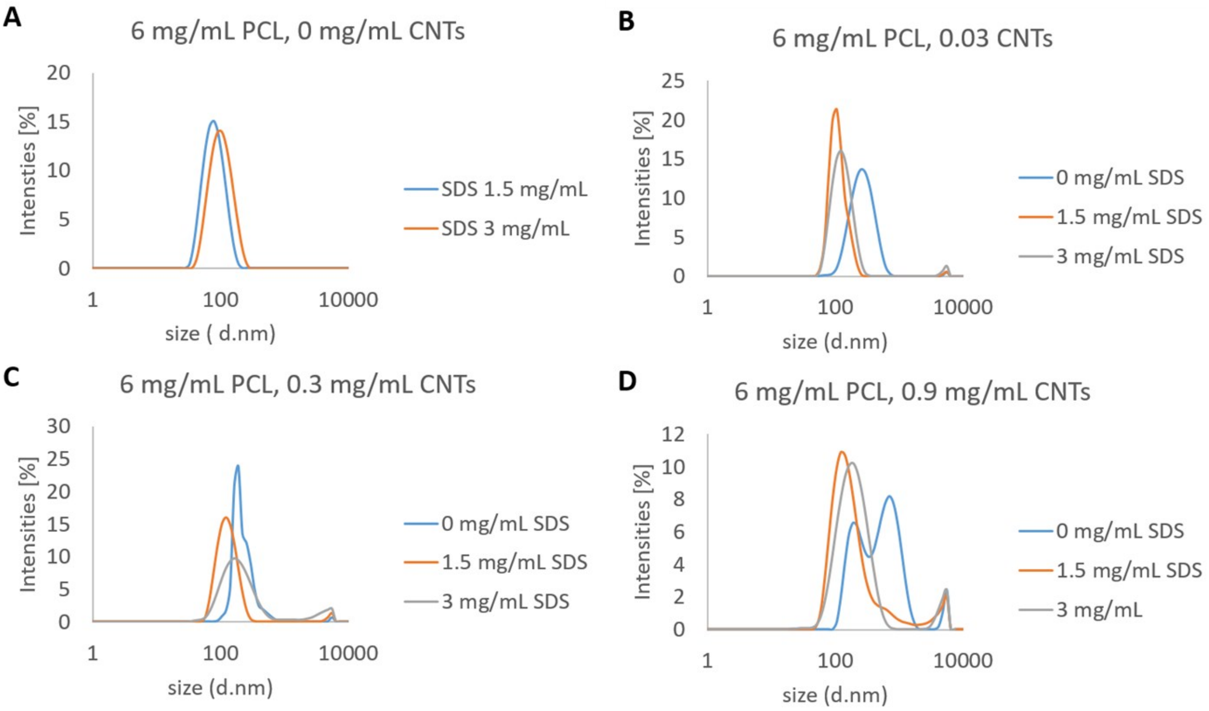

Figure S2.Intensity size distribution by DLS for samples of varying composition. A) Samples consisting of: 6mg/mL PCL, 0 mg/mL CNTs and either 1.5 or 3 mg/mL of SDS. B) Samples consisting of: 6 mg/mL PCL, 0.03 mg/mL CNTs and either 0, 1.5 or 3 mg/mL of SDS. C) Samples consisting of: 6 mg/mL PCL, 0.3 mg/mL CNTs and either 0, 1.5 or 3 mg/mL of SDS. D) Samples consisting of: 6 mg/mL PCL, 0.9 mg/mL CNTs and either 0, 1.5 or 3 mg/mL of SDS.

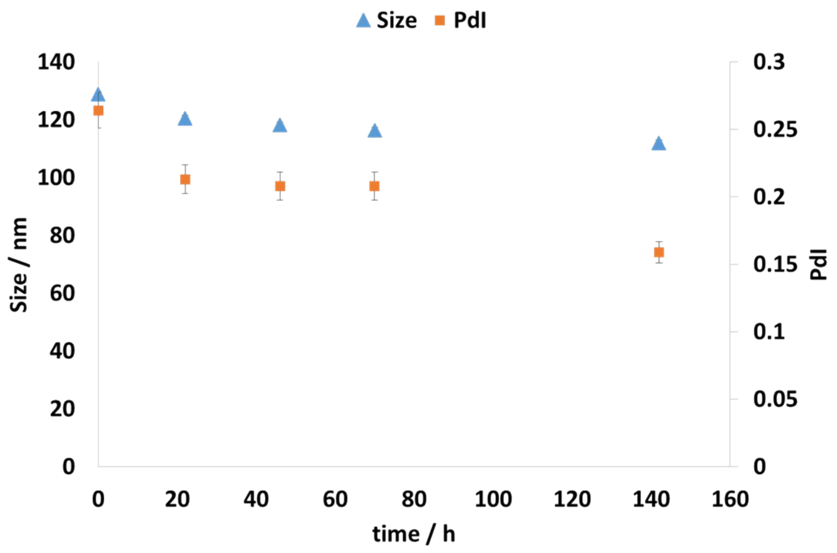

Figure S3. Graph shows size i.e. hydrodynamic diameters [nm] and polydispersity (PdI) versus time [h] obtained from DLS for samples in distilled water at 37 ˚C.

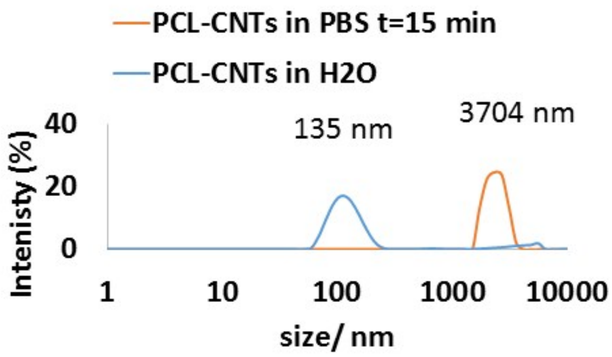

Figure S4. Graph shows size i.e. hydrodynamic diameters [nm] versus intensities [%] obtained from DLS for samples in distilled water and Phosphate buffer saline (PBS) incubated for 15 min.

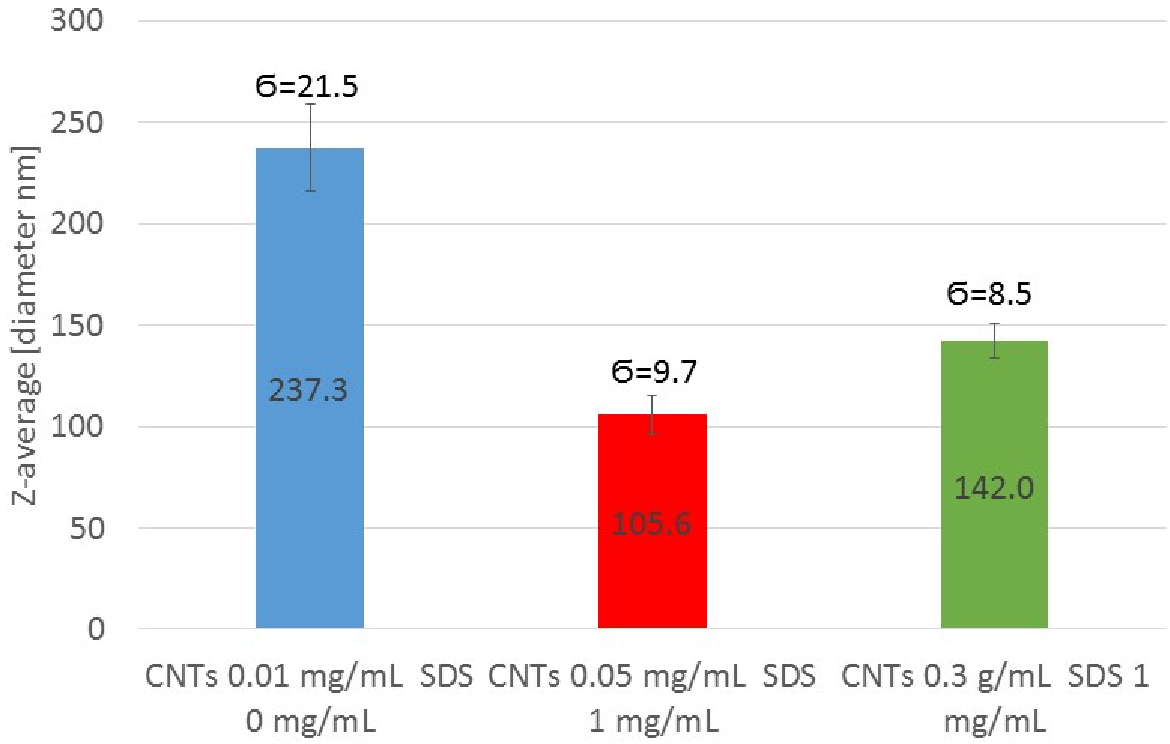

Figure S5. Example of results for reproducibility for samples obtained with 3 mg/mL of PCL. The synthesis was repeated 3 times.

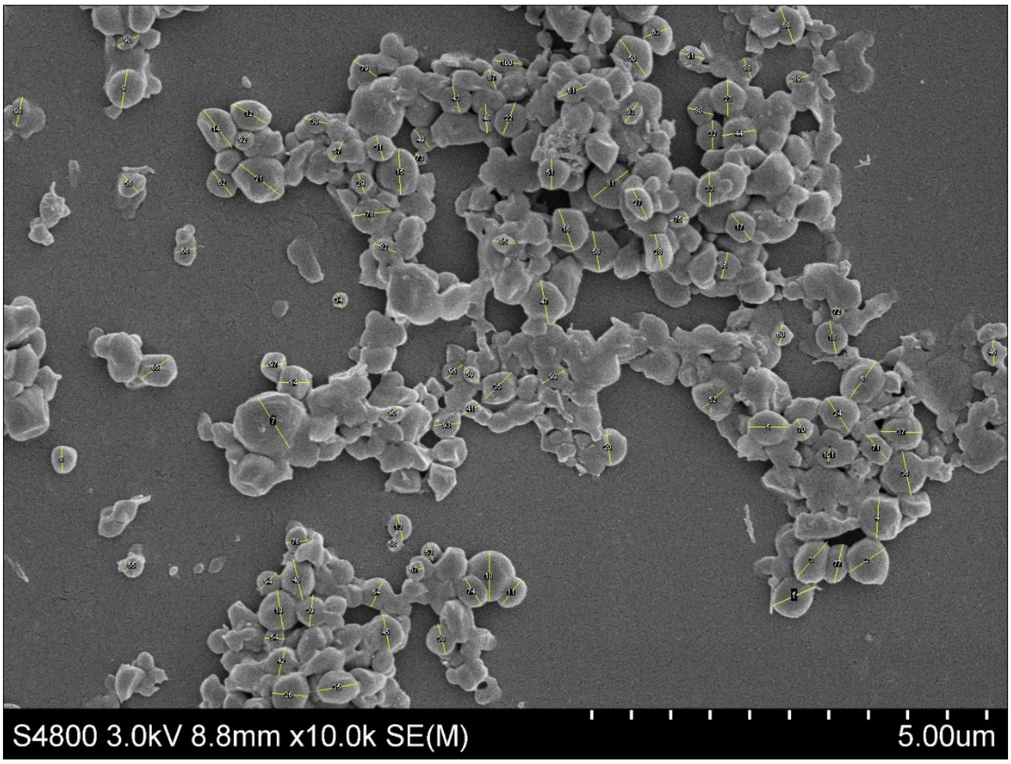

Figure S6. The picture presents the measurements of diameter for PCL particles obtained by ImageJ.

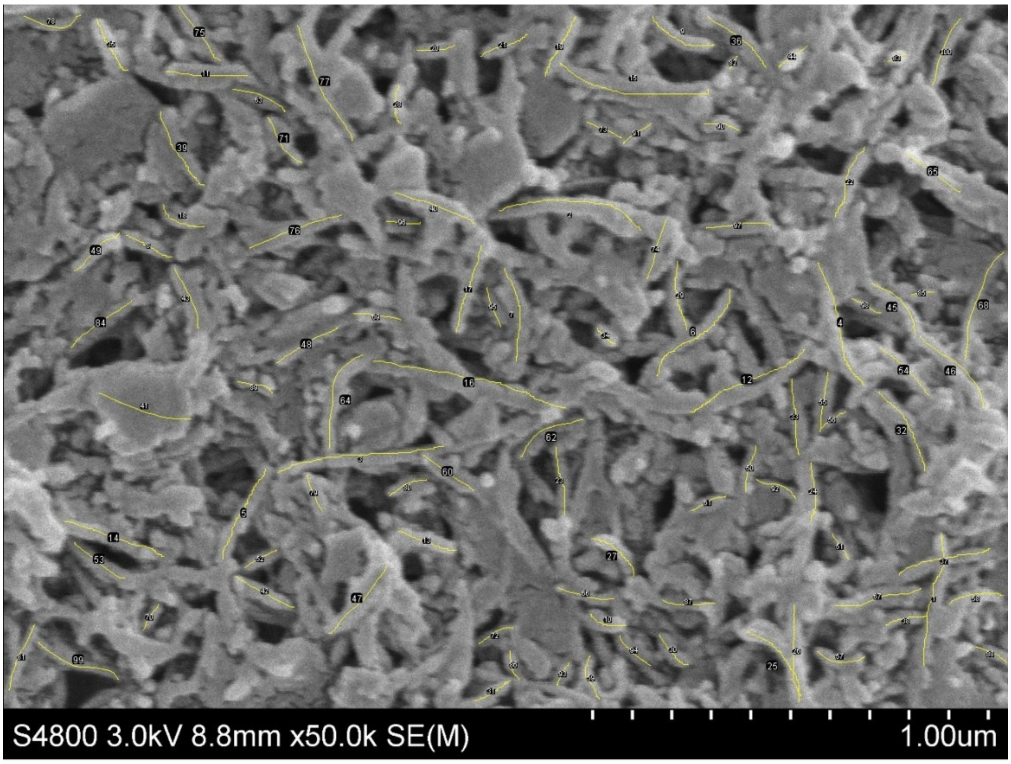

Figure S7. The picture presents the measurements of length PCL-CNTs particles obtained by ImageJ.

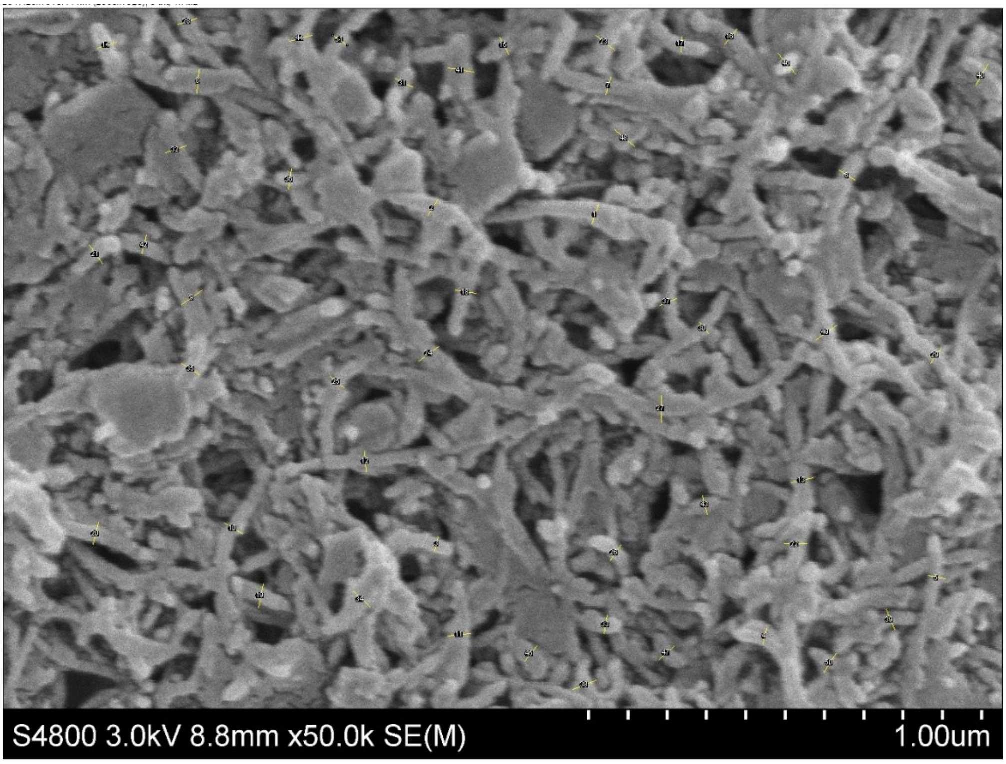

Figure S8. The picture presents the measurement of width for PCL-CNTs obtained by ImageJ.

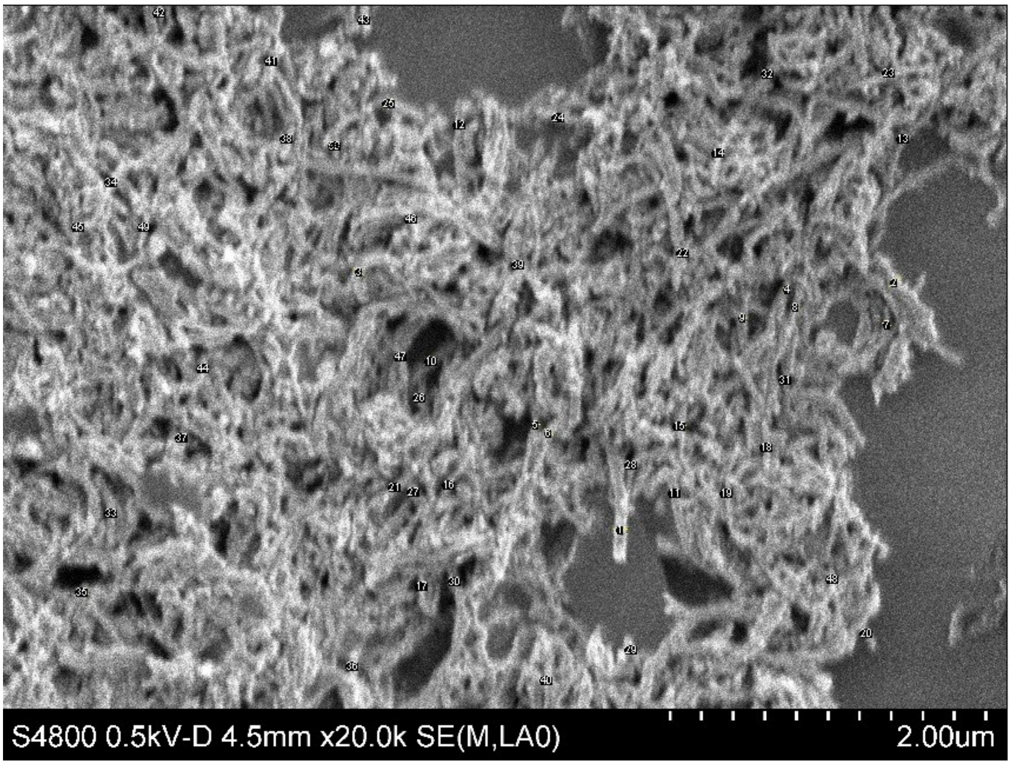

Figure S9. The picture presents the measurement of width for CNTs particles obtained by ImageJ.

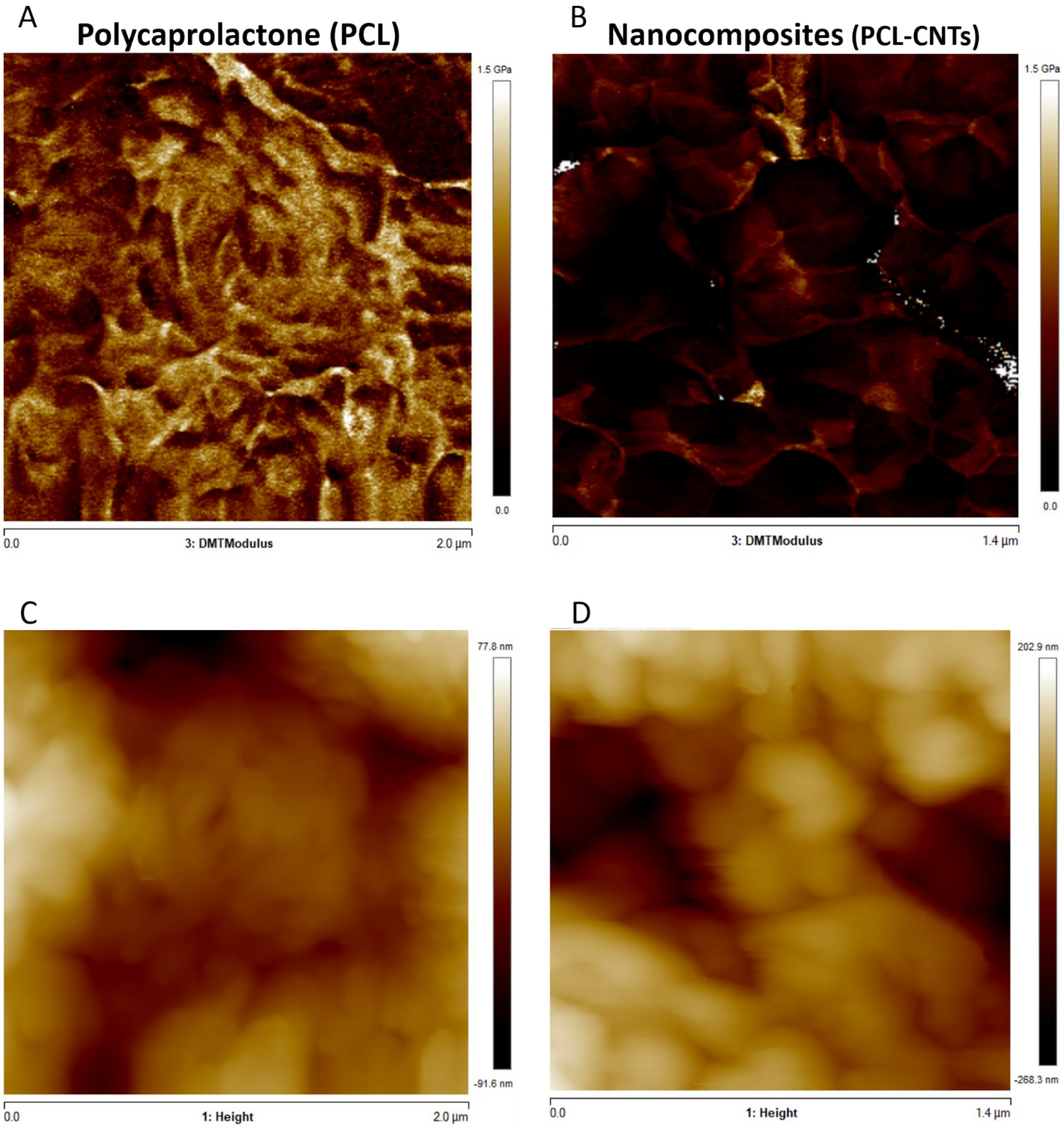

Figure S10. Analysis of the PCL and PCL-CNT nanocomposites by AFM to provide the DMT modulus and surface height. (A and B) show the DMT modulus for PCL and PCL-CNT nanocomposites. (C and D) show the heights for PCL and PCL-CNT nanocomposites.

Raman analysis and data

The sample preparation for Raman measurement was as follows; 1 mL of pre-prepared sample (dispersed at a concentration 1 mg/mL) was added onto a flat surface of aluminium foil and dried by exposure to air overnight. The Raman spectra were recorded on a BTC111-RAMAN-785 micro spectrometer with a built-in spectral 785 nm diode laser. A spectral coverage was measured in the range of 175-3100 cm^{-1}. Integration times: 30000 and average number of 6 measurements were conducted for each sample.

Raman measurements were conducted to characterise the samples. The presence of group peaks for PCL, CNTs and PCL-CNTs was checked. The Raman spectroscopy of multiwalled carbon nanotubes displayed two distinct graphite bands. The 1580 cm^{-1} (G band) assigned to the in-plane vibration of the C–C bond (G band) with a shoulder around 1604 cm^{-1}, typical of defective graphite-like materials and the band at 1342 cm^{-1} (D band) activated by the presence of disorder in carbon systems.[50] Analysis of the CNTs used in the nanocomposites matched the expected spectra, with the G band and D band observed. While, as expected the PCL alone sample did not show a G band (figure 11). This PCL sample displayed two characteristic peaks in the following regions: 1680 and 1480 cm^{-1}.[51] The PCL-CNTs nanocomposite was characterised by two main peaks i.e. 1580 and 1930 cm^{-1} and a weak peak at 1580 cm^{-1}. However the peaks 1580 cm^{-1} and 1680 cm^{-1} are very close to each other. The presence of the peaks for both the PCL and CNT indicates that the nanocomposite sample contains both of PCL and CNT.

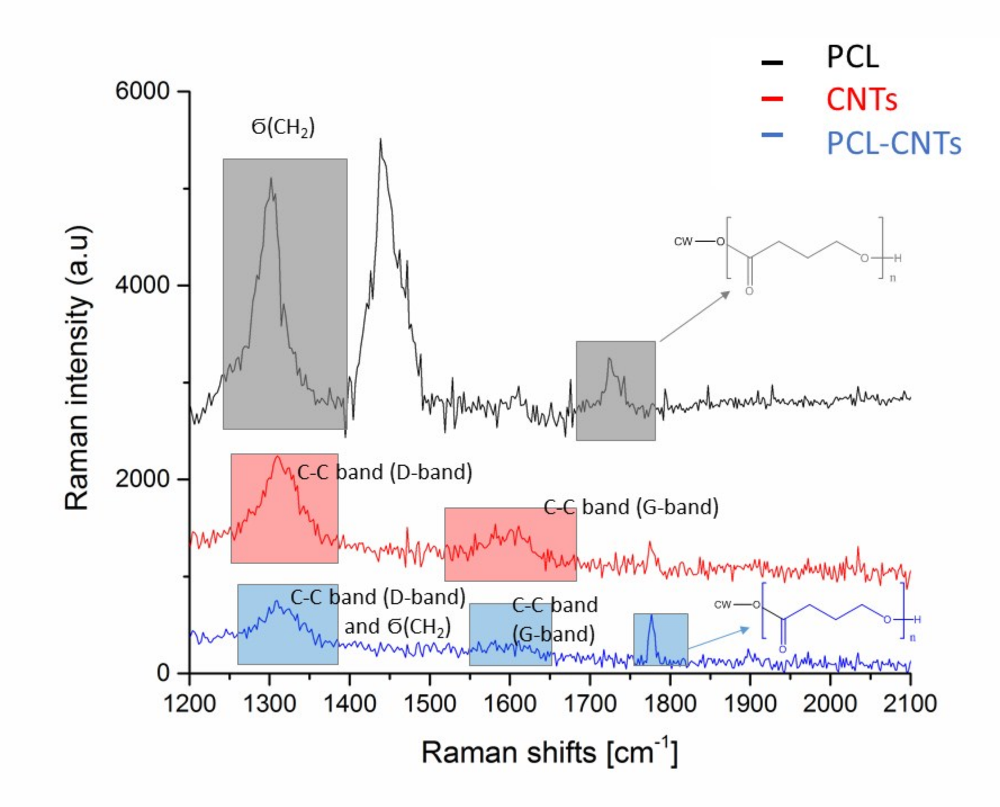

Figure S11. Raman spectra of PCL, CNTs and PCL-CNTs in the range 200-2000 cm^{-1}.

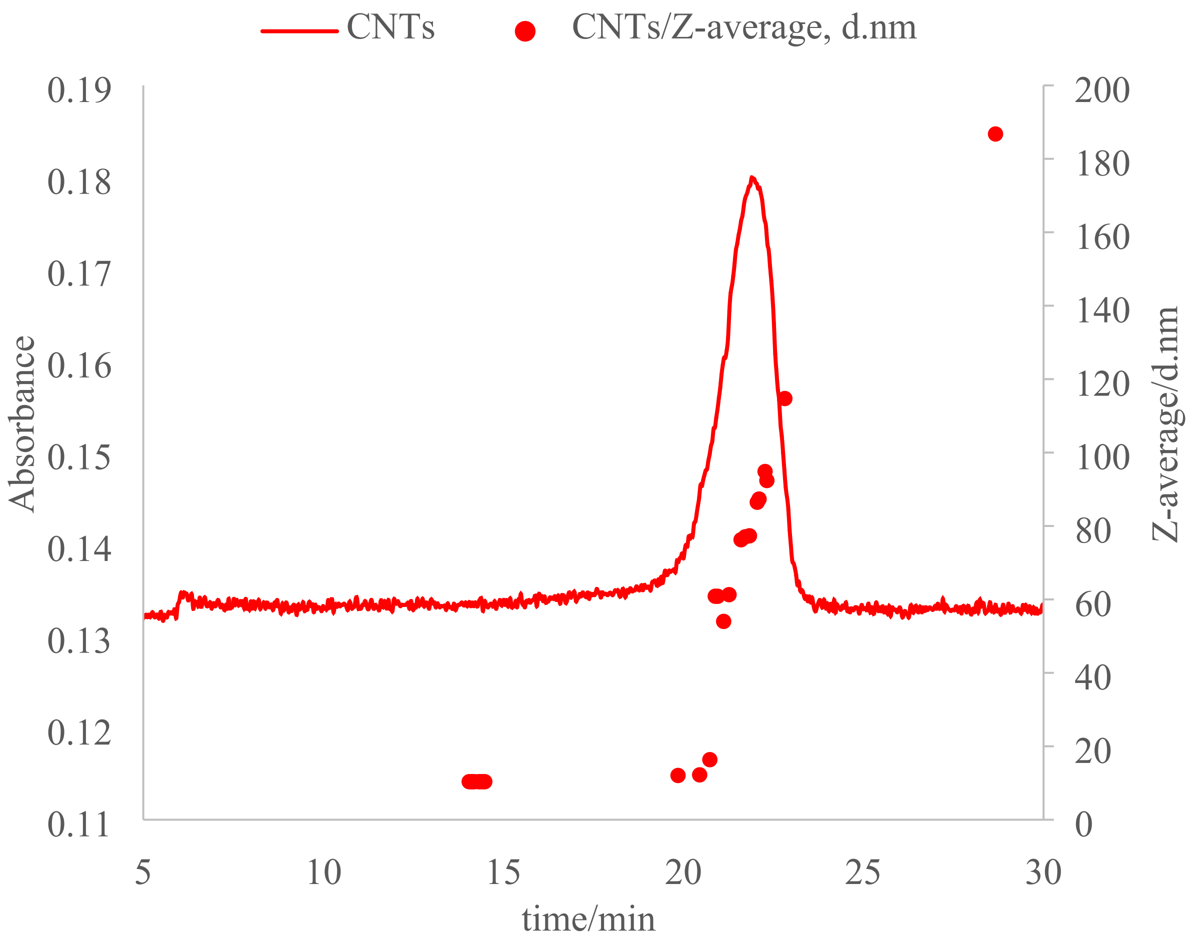

Figure S12. Fractogram of PCL, CNTs and PCL-CNTs obtained from asymmetric flow field flow fractionation coupled online with UV-VIS detector. Hydrodynamic diameters were obtained from DLS coupled online

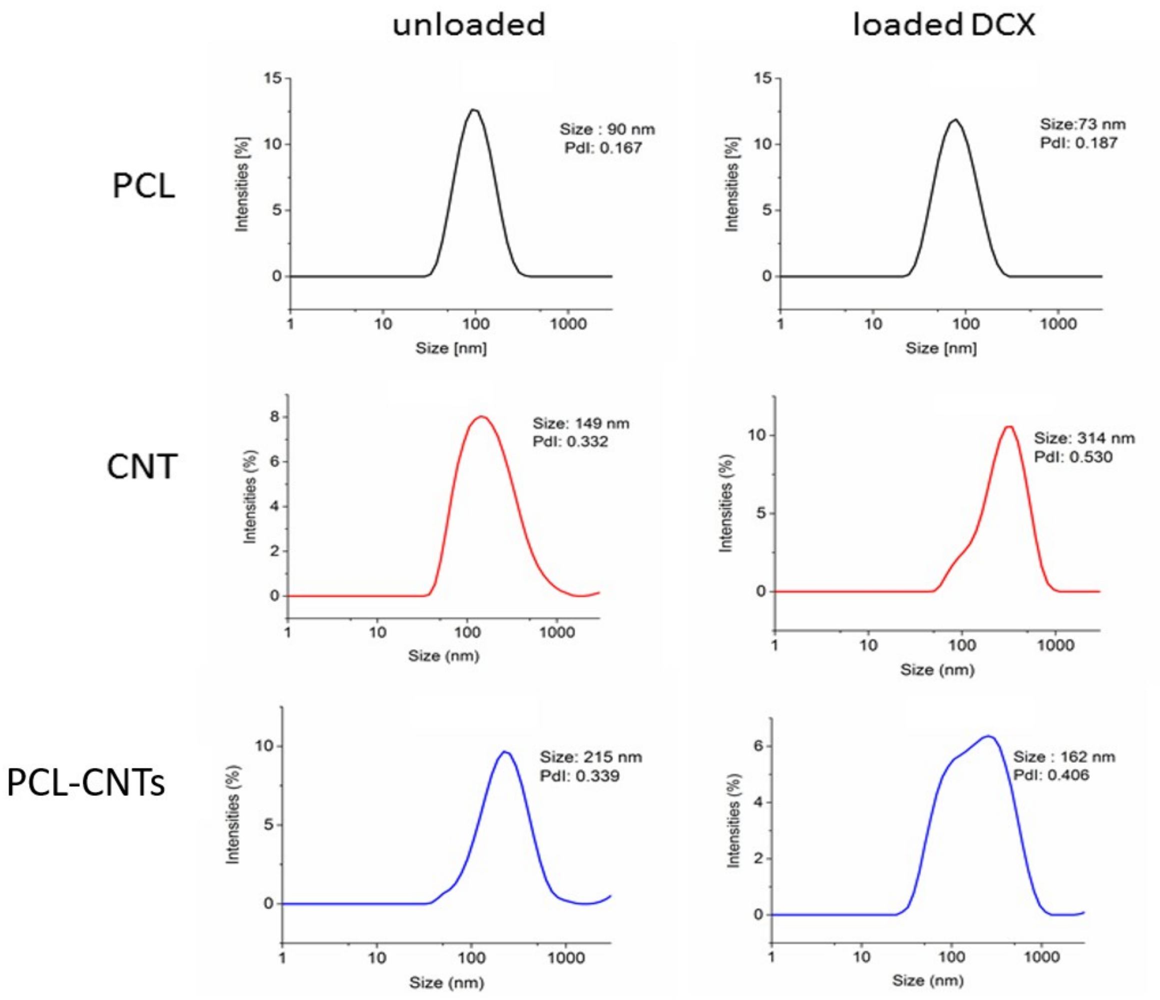

Figure S13. Graph shows size i.e. hydrodynamic diameters [nm] versus intensities [%] obtained from DLS for unloaded and loaded with DCX particles of PCL, CNTs and composites.

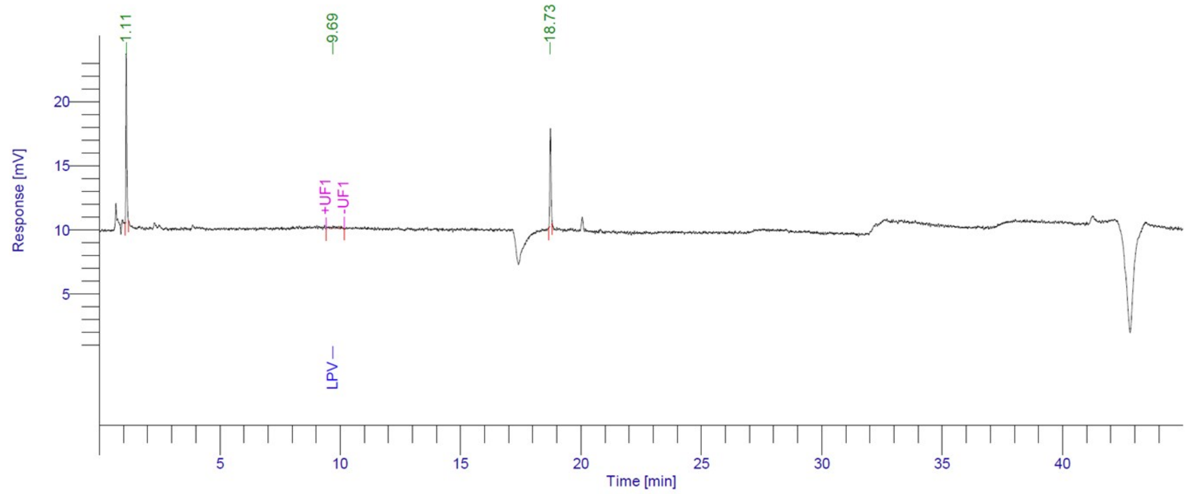

Figure S14. HPLC chromatogram of the released docetaxel from CNTs after 15 min.

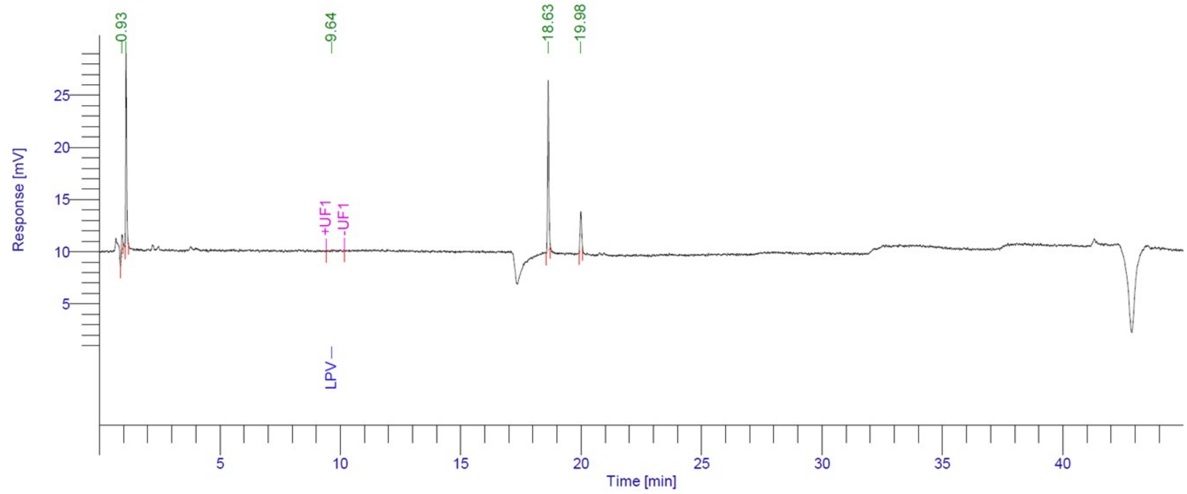

Figure S15. HPLC chromatogram of the released docetaxel from PCL-CNTs after 15 min.

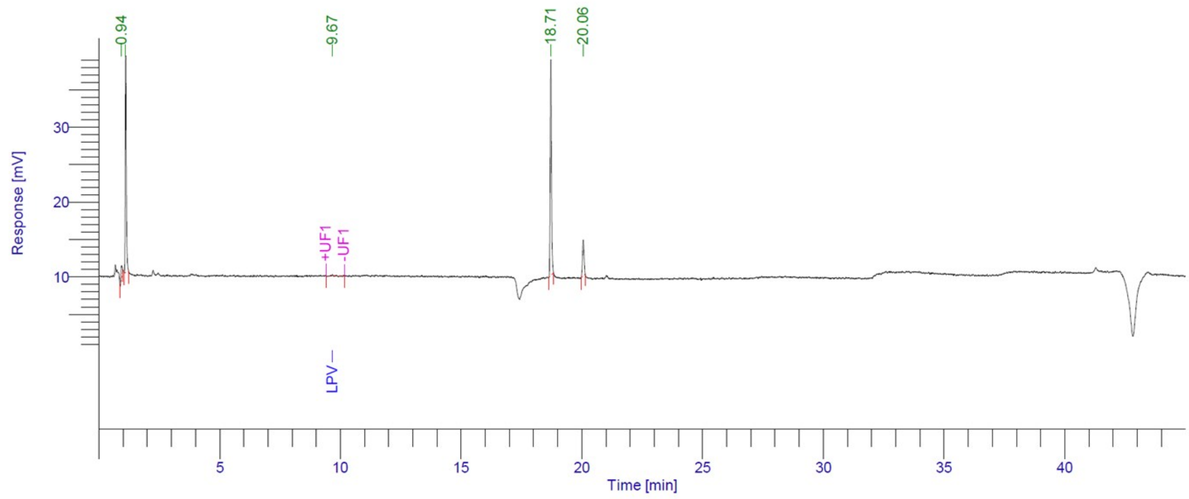

Figure S16. HPLC chromatogram of the released docetaxel from PCL after 15 min.

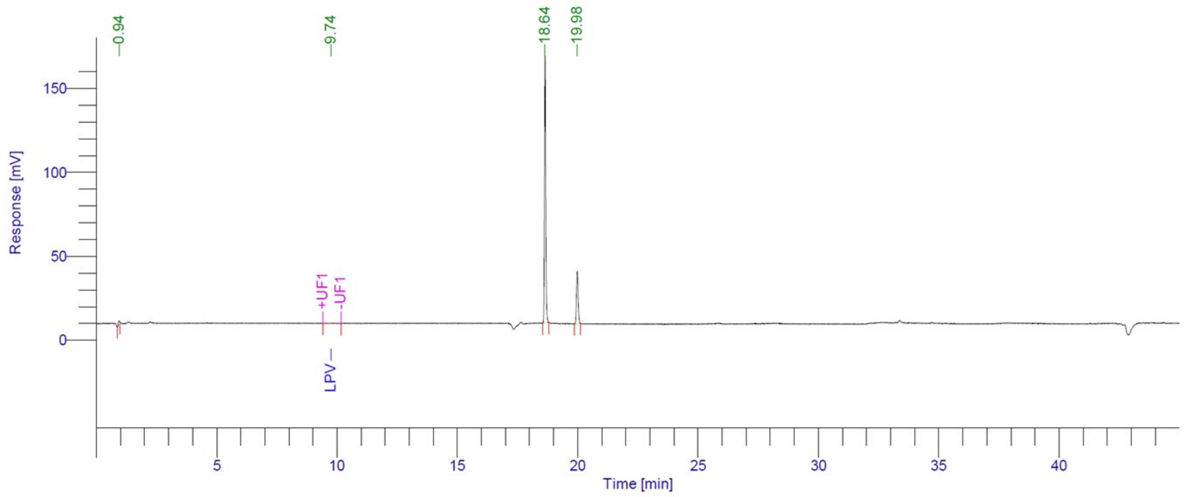

Figure S17. HPLC chromatogram of the standard docetaxel with the concentration 60 µg/mL.
